# Risk Factors associated with Paraurethral Duct Dilatation following Gonococcal Paraurethral Duct Infection in Men

**DOI:** 10.1371/journal.pone.0166355

**Published:** 2016-11-18

**Authors:** Wenge Fan, Qingsong Zhang, Lin Wang, Xun Ye, Tingwang Jiang

**Affiliations:** 1 Department of Dermatology, First People’s Hospital of Changshu City, Changshu Hospital Affiliated to Soochow University, Changshu 215500, Jiangsu Province, P. R. China; 2 Department of Dermatology, Traditional Chinese Medical Hospital of Changshu City, Changshu 215500, Jiangsu Province, P. R. China; 3 Department of Ultrasound, First People’s Hospital of Changshu City, Changshu Hospital Affiliated to Soochow University, Changshu 215500, Jiangsu Province, PR China; 4 Department of Clinical Laboratory, First People’s Hospital of Changshu City, Changshu Hospital Affiliated to Soochow University, Changshu 215500, Jiangsu Province, PR China; University of Texas Health Science Center at San Antonio, UNITED STATES

## Abstract

No studies have explored the risk factors for paraurethral duct dilatation following paraurethral duct infection by *Neisseria gonorrhoeae* in men undergoing ceftriaxone therapy. The present study was performed to explore the risk factors for paraurethral duct dilatation following paraurethral duct infection by *N*. *gonorrhoeae* in men undergoing ceftriaxone therapy and thus guide clinical interventions. We compared the demographic, behavioral, and clinical data of men with paraurethral duct infection by *N*. *gonorrhoeae* with and without dilatation of the paraurethral duct. Univariate analysis showed significant differences in age, disease course of the infected paraurethral duct, *Chlamydia trachomatis* infection in the paraurethral duct, and a history of paraurethral duct infection by *N*. *gonorrhoeae* between the patient and control groups *(P*<0.05). Multivariate logistic regression analysis showed consistent results (*P*<0.05). This study that shows delayed treatment may be a major risk factor for paraurethral duct dilatation secondary to paraurethral duct infection by *N*. *gonorrhoeae* in men. Age, *C*. *trachomatis* infection in the paraurethral duct, and a history of paraurethral duct infection by *N*. *gonorrhoeae* are also risk factors. Thus, educating patients to undergo timely therapy and treating the *C*. *trachomatis* infection may be effective interventions.

## Introduction

The paraurethral ducts in men are small, blind channels lined with columnar epithelium [1]. These ducts run parallel to the terminal part of the urethra for varying distances and open near or within the lips of the external meatus [2]. The paraurethral ducts appear to be embryological remnants and are not visible to the naked eye [3]. *Neisseria gonorrhoeae* can infect the paraurethral duct. Such infection clinically manifests as an erythematous swelling of the external urethral orifice. An abscess may form at the center of the swollen area and perforate outward to form a pinhead-like ostium at the opening of the paraurethral duct. Pressure on the glans penis can result in purulent excretion from this ostium.[4–8] High-frequency ultrasound can be used to examine the morphological features of the paraurethral duct after *N*. *gonorrhoeae* infection [9].This inflammation resolved with ceftriaxone in some patients in the present study, eliminating both the erythematous swelling of the external urethral orifice and the purulent excretion from the ostium. However, the ostium did not close, and overflow of transparent liquid was still visible after squeezing the lesion. The liquid was negative for all pathogens examined, and pathological biopsy of the lesion indicated the presence of paraurethral duct dilatation [10,11]. To better understand the risk factors for paraurethral duct dilatation following the paraurethral duct infection by *N*. *gonorrhoeae* in men, we performed a prospective case-control study in which we compared the demographic, behavioral, and clinical data of patients with paraurethral duct infection by *N*. *gonorrhoeae* with and without dilatation of the paraurethral duct.

## Patients and Methods

This study was approved by the Ethics Committee of the First People’s Hospital of Changshu City, Soochow University, Jiangsu Province, China. All participants provided written informed consent to participate in the study. We obtained written informed consent from the guardians of the minors included in the study.

### Inclusion criteria

All patients initially had local erythematous swelling at the external urethral orifice, with an ostium at its center; pressure caused expression of purulent excretion from the ostium, and *N*. *gonorrhoeae* was confirmed to be the pathogen. The morphological features of the paraurethral duct were examined using high-frequency ultrasound. The inclusion criteria for the control group were as follows: after ceftriaxone therapy, the local swelling at the external urethral orifice was resolved, the purulent excretion had disappeared, and the ostium was closed; pressure did not cause expression of fluid from the site where the ostium had been located; and there was no relapse during the 3-month follow-up. Thus, the condition was considered cured. The inclusion criteria for the patient group were as follows: after ceftriaxone therapy, the local erythematous swelling at the external urethral orifice was resolved and the purulent excretion had disappeared, but the ostium was not closed and pressure caused expression of transparent fluid from the ostium. However, testing for pathogens in the transparent liquid still showed negative results. The ostium did not close during the 3-month follow-up, and a diagnosis of paraurethral duct dilatation was made.

### Exclusion criteria

The exclusion criteria for both groups were as follows: local erythematous swelling was seen at the external urethral orifice, but the lesion did not perforate; two or more skin lesions were present; the lesion was not caused by *N*. *gonorrhoeae*; and the clinical data were incomplete.

### Laboratory tests

Paraurethral duct discharge was collected and examined under a microscope to detect intracellular Gram-negative diplococci within phagocytes. Specimens were also cultured to detect *N*. *gonorrhoeae*, *Ureaplasma urealyticum*, or other bacteria, and polymerase chain reaction was used to detect the DNA of *N*. *gonorrhoeae*, *Chlamydia trachomatis*, *U*. *urealyticum*, and herpes simplex virus type 1 or 2. Venous blood samples were taken and analyzed using the rapid plasma reagin test, *Treponema pallidum* hemagglutination assay, and human immunodeficiency virus antibody assay.

### Data collection

The demographic, behavioral, and clinical data from patients with paraurethral duct infection by *N*. *gonorrhoeae* were collected. These data included age, marital status, educational background, source of infection, sexual orientation, sexual behavior pattern, condom use, prepuce condition, morphological features of the paraurethral duct infected by *N*. *gonorrhoeae* (diameter and length of the paraurethral duct), disease course of the paraurethral duct infected by *N*. *gonorrhoeae* (amount of time the patient had experienced symptoms before undergoing treatment), *C*. *trachomatis* infection of the paraurethral duct, whether the patient drank alcohol or engaged in sexual intercourse during paraurethral duct infection by *N*. *gonorrhoeae*, history of paraurethral duct infection by *N*. *gonorrhoeae*, and concomitant diseases.

### Statistical analysis

All data analyses were performed using SPSS 13.0 for Windows (IBM Corporation, Chicago, IL, USA), and *P<*0.05 was considered statistically significant. Continuous variables were analyzed using Student’s *t*-test, and discrete variables were compared using the chi-square test. Discrete variables that were not feasible for analysis by the chi-square test were analyzed using Fisher’s exact probability test. Multivariate logistic regression analysis was applied to analyze the risk factors for secondary paraurethral duct dilatation. The variables for logistic regression analysis were screened using a stepwise method, and the 95% confidence intervals were calculated.

## Results

**Fig 1:**
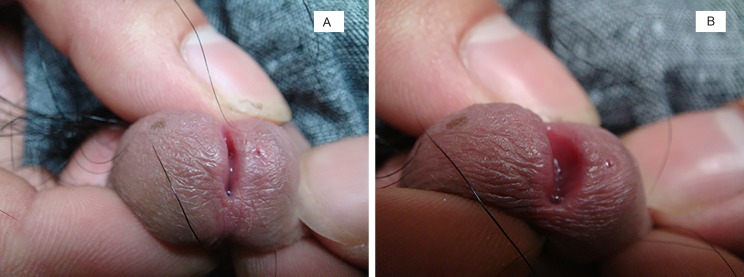
**(A).** A pinhead-like ostium was present at the 3 o’clock position on the left side of the external urethral orifice. (**B).** An overflow of transparent liquid was visible after squeezing the lesion.

In total, 106 male patients with paraurethral duct infection by *N*. *gonorrhoeae* were diagnosed and treated in our department from October 1997 to May 2015. Serological tests for syphilis and human immunodeficiency virus were negative for all patients. All patients were treated with intramuscular ceftriaxone sodium (1 g once daily for 5 days). Patients with concomitant chlamydial urethritis were simultaneously treated with azithromycin (0.5 g once daily for 5 days). After treatment, paraurethral duct dilatation was found in 15 patients (14.15%) (Fig 1). These 15 patients were included in the patient group, and 71 patients were included in the control group. The age of patients in the patient group was 28 to71 years (mean, 44.9 ± 12.0 years), and that of patients in the control group was 16 to70 years (mean, 30.8 ± 13.2 years; t = 3.8, *P<*0.01). There were no significant differences in the other demographic and behavioral features between the two groups (Table 1). In the patient group, the paraurethral ducts had a diameter of 0.7 to1.2 mm (mean, 1.1± 0.2 mm) and a length of 7.5 to11.2 mm (mean, 8.5 ± 1.2 mm). In the control group, the diameter and length were 0.7 to1.4 mm (mean, 1.2 ± 0.2 mm) and 7.1 to12.7 mm (mean, 8.4 ± 1.3 mm), respectively. The diameter (t = −1.74, *P* = 0.09) and length (t = 0.27, *P* = 0.79) were not significantly different between the two groups. The disease course ranged from 6 to 35 days (mean, 15.6 ± 6.5 days) in the patient group and from 1 to 16 days (mean, 4.9 ± 2.8 days) in the control group (t = 10.2, *P<*0.01). The disease course for both groups was divided into three grades for logistic regression: ≤ 7 days, 8 to 14 days, and ≥15 days. In addition, *C*. *trachomatis* infection of the paraurethral duct, a history of paraurethral duct infection by *N*. *gonorrhoeae* and disease course were also significantly different between these two groups (Table 1).

**Table 1:** Comparison of demographic, behavioral, and clinical data between patient and control groups.

Variables	Case group (n = 15) n (%)	Control group (n = 71) n (%)	χ2 value	*P* value
**Age (years)**			5.046	0.02
<45	6 (40.00)	50 (70.42)		
≥45	9 (60.00)	21 (29.58)		
**Marital status**			0.0983	0.76
Unmarried	4 (26.67)	19 (26.76)		
Married	11 (73.33)	52 (73.24)		
**Education background**			0.03	0.87
Middle or high school	6 (40.00)	30 (42.25)		
College	9 (60.00)	41 (57.75)		
**Source of infection**				0.54
Use of sex workers	14 (93.33)	68 (95.77)		
Others	1 (6.67)	3 (4.23)		
**Sexual orientation**				
Heterosexual	15 (100)	71 (100)		
Homosexual	0 (0)	0 (0)		
Bisexual	0 (0)	0 (0)		
**Sexual behavior pattern**				
Genital/genital	15 (100)	71 (100)		
Genital/oral	0 (0)	0 (0)		
Genital/anal	0 (0)	0 (0)		
**Condom use**			0.0076	0.98
Never	12 (80.00)	59 (83.10)		
Sometimes	3 (20.00)	12 (16.90)		
**Prepuce condition**			0.0451	0.71
Normal	12 (80.00)	58 (81.69)		
Redundant prepuce	3 (20.00)	13 (18.31)		
**Drinking alcohol during the paraurethral duct infected by *gonococcus***			0.0841	0.78
Yes	2 (13.33)	5 (7.04)		
No	13 (86.67)	66 (92.96)		
**Sexual intercourse during the paraurethral duct infected by *gonococcus***			0.2559	0.69
Yes	2 (13.33)	4 (5.63)		
No	13 (86.67)	67 (94.37)		
***C*. *trachomatis* infection in the paraurethral duct**			7.490	0.01
Yes	4 (26.67)	2 (2.82)		
No	11 (73.33)	69 (97.18)		
**Previous history of the paraurethral duct infected by *gonococcus***			5.610	0.02
Yes	4 (26.67)	3 (4.23)		
No	11 (73.33)	68 (95.77)		
**Concomitant diseases**				0.06
With diabetes	2 (13.33)	1 (1.41)		
Without diabetes	13 (86.67)	70 (98.59)		
**Course of disease**				<0.01
≤ 7 days	2 (13.33)	46 (64.79)		
8 to 14 days	4 (26.67)	22 (30.99)		
≥15 days	9(60.00)	3(4.22)		

After step-by-step screening with logistic regression, the following risk factors were identified: age, disease course, *C*. *trachomatis* infection of the paraurethral duct, a history of paraurethral duct infection by *N*. *gonorrhoeae* and concomitant disease. The first four risk factors were significant risk factors for secondary paraurethral duct dilatation after multivariate logistic regression analysis (Table 2).

**Table 2:** Results of logistic regression for identification of significant risk factors.

Parameter	Odds ratio	95%CL for OR	P-value
Age	2.46	1.33–6.39	0.03*
*Chlamydia trachomatis* infection in the paraurethral duct	3.96	1.69–8.75	0.01*
Previous *gonococcus* infection of paraurethral duct	2.19	1.22–7.48	0.03*
Concomitant disease (diabetes)	1.12	0.65–1.79	0.46
Disease course ^a^	6.25	2.76–13.26	0.00**

*P<0.05

**P<0.01

a, amount of time the patient had experienced symptoms before undergoing treatment

## Discussion

Infection of the paraurethral duct by *N*. *gonorrhoeae* is a localized complication in male patients with gonorrhea. In total, 7058 male patients with gonorrhea that was confirmed both clinically and by laboratory test results were treated in our department from October 1997 to May 2015, and 106 (1.50%) of these patients had accompanying paraurethral duct infection by *N*. *gonorrhoeae*. A prolonged disease course, sexual intercourse during gonorrhea, repeated squeezing of the penis, and a redundant prepuce may be risk factors for paraurethral duct infection by *N*. *gonorrhoeae* in male patients with gonorrhea [6]. Paraurethral duct dilatation is a sequela of paraurethral duct infection by *N*. *gonorrhoeae* and has characteristic clinical, histopathological, and high-frequency ultrasound findings. Wedge excision is an effective treatment for this condition [10].

In the current study, univariate analysis showed that age, disease course, infection of the paraurethral duct with *C*. *trachomatis*, and a history of paraurethral duct infection by *N*. *gonorrhoeae* were significantly different between the patient and control groups. Multivariate logistic regression analysis showed consistent results. A prolonged disease course and repeated infection in male patients with paraurethral duct infection by *N*. *gonorrhoeae* may cause the acute inflammation to become chronic. Research using rat models has shown that chronic prostate inflammation can cause fibrosis of the prostate and its surrounding tissues [12]. Additionally, controlling chronic prostate inflammation can, to some extent, reverse the progression of fibrosis [13]. Studies of the human prostate have reached similar conclusions [14,15]. Some authors have also found that urethral fibrosis reduces urethral compliance [16]. Therefore, the pathogenic mechanism of paraurethral duct dilatation may be as follows: a prolonged disease course causes acute inflammation to become chronic, and the paraurethral duct thus becomes more susceptible to fibrosis. As a result, the compliance of the paraurethral duct wall decreases or is even eliminated, and the dilated paraurethral duct cannot close by itself. Therefore, educating patients to receive timely treatment may be an effective intervention.

Paraurethral duct inflammation is most commonly caused by *N*. *gonorrhoeae* infection, but it can also be a consequence of infection with *C*. *trachomatis* or other pathogens [7,17]. Coinfection with *N*. *gonorrhoeae* and *C*. *trachomatis* in the paraurethral duct can exacerbate the inflammation because the synergetic effect between *N*. *gonorrhoeae* and *C*. *trachomatis* induces a high level of bacterial multiplication that aggravates the infection by *N*. *gonorrhoeae* [18].

The pathologic changes caused by repeated and persistent infection with *C*. *trachomatis* may result in tissue damage secondary to chronic inflammation [19]. Previous studies have confirmed that chronic *C*. *trachomatis* infection can induce inflammatory damage and fibrosis in genital ducts such as the oviduct [20]. Furuya et al.[21] also found that *C*. *trachomatis* infection can induce ectasia of the seminal vesicles, which have an epithelial structure similar to that of the paraurethral ducts. Thus, paraurethral duct fibrosis and dilation can be attributed to inflammation caused by *C*. *trachomatis*, and treating the *C*. *trachomatis* coinfection may be an effective intervention.

Age may contribute to the degeneration of physiological function in patients with paraurethral duct dilatation. One study showed that epithelial regression with senility was the major risk factor for deferent duct dilation and cystogenesis [22]. Thus, regression of the paraurethral duct epithelium may induce post-infection dilation of the paraurethral duct.

The incidence of paraurethral duct infection by *N*. *gonorrhoeae* is low in male patients, and paraurethral duct dilatation secondary to this disease is rare in the clinical setting. In our department, only 15 patients with complete data were identified within the past 18 years. Multicenter studies with larger sample sizes may identify more risk factors for paraurethral duct dilatation.

## Supporting Information

S1 Dataset Raw material of manuscript. (XLS)
